# Evaluation of transplantation sites for human intestinal organoids

**DOI:** 10.1371/journal.pone.0237885

**Published:** 2020-08-27

**Authors:** Akaljot Singh, Holly M. Poling, Nambirajan Sundaram, Nicole Brown, James M. Wells, Michael A. Helmrath

**Affiliations:** 1 Division of Developmental Biology, Cincinnati Children's Hospital Medical Center, Cincinnati, OH, United States of America; 2 Division of Pediatric General and Thoracic Surgery, Cincinnati Children's Hospital Medical Center, Cincinnati, OH, United States of America; 3 Department of Surgery, University of Cincinnati, Cincinnati, OH, United States of America; Università degli Studi della Campania, ITALY

## Abstract

Our group has developed two transplantation models for the engraftment of Human Intestinal Organoids (HIOs): the renal subcapsular space (RSS) and the mesentery each with specific benefits for study. While engraftment at both sites generates laminated intestinal structures, a direct comparison between models has not yet been performed. Embryonic stem cells were differentiated into HIOs, as previously described. HIOs from the same batch were transplanted on the same day into either the RSS or mesentery. 10 weeks were allowed for engraftment and differentiation, at which time they were harvested and assessed. Metrics for comparison included: mortality, engraftment rate, gross size, number and grade of lumens, and expression of markers specific to epithelial differentiation, mesenchymal differentiation, and carbohydrate metabolism. Mortality was significantly increased when undergoing mesentery transplantation, however engraftment was significantly higher. Graft sizes were similar between groups. Morphometric parameters were similar between groups, however m-tHIOs presented with significantly fewer lumens than k-tHIO. Transcript and protein level expression of markers specific to epithelial differentiation, mesenchymal differentiation, and carbohydrate metabolism were similar between groups. Transplantation into both sites yields viable tissue of similar quality based on our assessments with enhanced engraftment and a dominant lumen for uniform study benefiting the mesenteric site and survival benefiting RSS.

## Introduction

Our groups previously developed a protocol for the development of Human Intestinal Organoids (HIOs) from embryonic stem cells and induced pluripotent stem cells [1, 2]. When employing a transplantation strategy, the structural organization and tissue complexity of an HIO is markedly enhanced [3]. These transplanted HIOs (tHIOs) present with crypts, villi, and mesenchymal structures, such as differentiated smooth muscle layers, making them a powerful model of the human gut [3, 4].

Initially, transplants were performed in the renal subcapsular space (RSS), because this location was proven a successful engraftment site for pancreatic islets [5]. Murine kidneys are highly vascularized, readily accessible, and the transplanted materials are easy to identify upon engraftment. In later studies, we expanded our transplantation strategies to include a mesenteric site to provide a similar anatomical location as native intestinal tissue [4, 6]. This site also offers advantages when considering downstream surgical applications and techniques including a procedure we have termed the “Tie In,” in which the tHIO can be put into continuity with the host gut [6]. Importantly, the mesentery shares a blood supply with the intestine, and is thought to contribute to intestinal function, including peristalsis, immune function, and tissue repair [7]. While our previous studies have demonstrated that the mesentery is a viable alternative site for HIO transplantation, a direct comparison between the two transplantation sites has not been performed [8]. Here, we aim to characterize potential similarities and differences between tHIOs engrafted into both sites.

## Methods

### Animals

Male immunodeficient nonobese diabetic (NOD) severe combined immunodeficiency (SCID) interleukin-2Rγnull (NSG) mice aged between eight and ten weeks were used in all transplantation experiments. Mice were housed in the pathogen-free animal vivarium of Cincinnati Children's Hospital Medical Center (CCHMC). Handling was performed humanely in accordance with the NIH Guide for the Care and Use of Laboratory Animals. NSG mice were fed antibiotic chow (275p.p.m. sulfamethoxazole and 1365p.p.m.trimethoprim; Test Diet). Both food and water were provided ad libitum pre- and post-operatively. All experiments were performed with the prior approval of the Institutional Animal Care and Use Committee of CCHMC (Signaling Pathways associated with Intestinal Stem Cell Expansion, Protocol No. 2018–0092).

### Generation of HIOs

HIOs were generated and maintained as previously described [1, 2]. Briefly, H1 embryonic stem cells (WiCell Research Institute, Inc.) were grown in feeder-free conditions in Matrigel (BD Biosciences) coated plates and maintained with mTESR1 media (Stem Cell Technologies). For induction of definitive endoderm (DE), cells were plated at a density of 65,000–75,000 cells per well in 24-well plates. Cells were allowed to grow for two days before treatment with 100 ng/ml of Activin A for three days as previously described. DE was then treated with hindgut induction medium (RPMI 1640, 100x NEAA, 2% dFCS), for four days with 100 ng/ml FGF4 (R&D) and 3 μM Chiron 99021 (Tocris) to induce the formation of spheroids. Spheroids were then collected and plated in Growth Factor Reduced (GFR) Matrigel and maintained in intestinal growth medium (Advanced DMEM/F-12, N2 supplement, B27 supplement, 15 mM HEPES, 2 mM L-glutamine, penicillin-streptomycin) supplemented with 100 ng/ml EGF (R&D) to generate human intestinal organoids (HIOs). Thereafter, media was changed twice a week. HIOs were replated in fresh Matrigel after 14 days. HIOs were utilized for surgical transplantation on day 28.

### Transplantation of human intestinal organoids

A single HIO matured in vitro for 28 days was transplanted into either the kidney capsule or mouse mesentery. Due to concerns for animal wellbeing, no single animal received grafts in both sites, as both surgeries are considered major surgical procedures. Preparations for both placements were similar. Mice were anesthetized with 2% inhaled isoflurane (Butler Schein) and the flank or abdominal wall was shaved, and prepped in a sterile fashion with isopropyl alcohol and povidine-iodine. Double layer incision closures were also performed for both sites. Upon closing, all mice were given a single injection of long acting Buprenex (0.05 mg/kg; Midwest Veterinary Supply) for pain management. Survival of mice was followed out to 10 weeks at which time the mice were humanely euthanized.

### Renal subcapsular transplantation

This procedure was performed as previously described [3, 6]. Briefly, a 1 cm posterior subcostal skin incision was made, followed by a retroperitoneal muscle incision to expose the kidney. The kidney was removed from the cavity and a subcapsular pocket created large enough for HIO insertion. The HIO was implanted securely within the subcapsular pocket. Then, the kidney was returned to the peritoneal cavity and incision closed.

### Mesentery transplantation

This procedure was performed as previously described [4, 6]. Briefly, a 2 cm midline incision was made to gain access to the abdominal cavity. The cecum was identified and removed from the body cavity with the intestine following. The mesentery was splayed out, and an appropriate location with bifurcating mesenteric vessels identified as the transplantation site. Here, octyl/butyl cyanoacrylate adhesive glue (GLUture, Abbott Laboratories) was applied and the HIO seeded. After allowing the glue to cure, the bowel was returned within the abdominal cavity and incision closed.

### Gross measurement of HIOs

Harvested tHIOs were photographed alongside a metric ruler and analyzed using ImageJ (NIH). For each image, the tHIO was measured widthwise and a 1 mm measurement on the ruler made in the same image was used as a conversion factor.

### Tissue processing, immunohistochemistry, and microscopy

Transplanted HIOs were harvested and divided in half. Half of each graft was fixed overnight in 4% paraformaldehyde at 4°C, processed and embedded in paraffin (the other half was flash frozen and used for gene expression analysis). Slides of 5 μm thick tissue sections were used for staining. Slides were deparaffinized, rehydrated and antigen retrieval performed. Incubation for both primary and secondary antibodies took place at 4°C overnight. Antibodies and their respective dilutions are listed in Table 1. Images were captured on a Nikon Eclipse Ti and analyzed using Nikon Elements Imaging Software (Nikon).

**Table 1:** Antibody information for immunofluorescence staining.

	Antigen	Dilution	Host	Company: Catalog Number
**Primary**	alpha-2, Smooth Muscle Actin (ACTA2)	1:400	Mouse	Abcam: ab7817
	Chromogranin A (CHGA)	1:500	Mouse	DSHB: CPTC-CHGA-1
	Dipeptidylpeptidase 4 (DPPIV)	1:1000	Goat	R&D: AF954
	E-Cadherin (CDH1)	1:300	Mouse	BD: 610182
	Elastin Microfibril Interfacer 1 (EMILIN1)	1:400	Rabbit	Atlas: HPA002822
	Lysozyme (hLYZ)	1:2000	Rabbit	Biorad: 0100–0523
	Marker Of Proliferation Ki-67 (MKI67)	1:350	Rabbit	Thermo Fisher: RM-9106-50
	Mucin-2 (MUC2)	1:1100	Rabbit	Abcam: ab134119
	Olfactomedin 4 (OLFM4)	1:200	Mouse	Cell Signalling: 14369
	Sucrase-Isomaltase (SI)	1:800	Rabbit	Sigma: HPA011897
	Vimentin (VIM)	1:350	Goat	Santa-Cruz: SC-7557
**Secondary**	α-mouse AF568	1:1000	Donkey	Life Technologies: A10037
	α-rabbit AF647	1:1000	Donkey	Life Technologies: A31573
	α-rabbit AF488	1:1000	Donkey	Life Technologies: A21206

### RNA isolation and RT-qPCR

At the time of harvest, half of each graft was flash frozen in liquid nitrogen. RNA was extracted from flash frozen samples using RNeasy Plus Micro Kit (Qiagen) according to manufacturer’s protocols. Subsequently, a cDNA reverse transcription kit (Applied Biosystems) was used for cDNA synthesis. Taqman (Applied Biosystems) gene expression assays were then performed in triplicate using a OneStep thermocycler (Applied Biosystems). Beta-Actin (ACTB) was used as a reference. TaqMan probes are listed in Table 2.

**Table 2:** TaqMan probe information for transcript analysis.

Gene	Gene Name	Gene ID	TaqMAN#	Accession #	Amplicon Length (bp)	Marker Type
ACTA2	alpha-2, Smooth Muscle Actin	59	Hs00426835_g1	NM_001141945.1	105	Smooth Muscle
ACTB	Beta-actin	60	Hs01060665_g1	NM_001101.3	63	Housekeeping
CHGA	Chromogranin A	1113	Hs00900375_m1	NM_001275.3	88	Enteroendocrine Cell
DPPIV	Dipeptidylpeptidase 4	1803	Hs00175210_m1	NM_001935.3	90	Enterocyte
EMILIN1	Elastin Microfibril Interfacer 1	11117	Hs00918337_g1	NM_007046.3	65	Mesenchyme
EPCAM	Epithelial Cell Adhesion Molecule	4072	Hs00901885_m1	NM_002354.2	95	Epithelium
LGR5	Leucine Rich Repeat Containing G Protein-Coupled Receptor 5	8549	Hs00969422_m1	NM_001277226.1	61	Intestinal Stem Cell
LYZ	Lysozyme	4069	Hs00426232_m1	NM_000239.2	67	Paneth Cell
OLFM4	Olfactomedin 4	10562	Hs00197437_m1	NM_006418.4	85	Intestinal Crypt
SI	Sucrase-Isomaltase	6467	Hs00356112_m1	NM_001041.3	64	Enterocyte
VIM	Vimentin	7431	Hs00185584_m1	NM_003380.3	73	Fibroblast

### Data representation and statistical analysis

All bar graphs indicate the mean ± standard deviation. GraphPad Prism software (San Diego, CA, USA) was used to perform Student’s t-test and Wilcoxon-Mann-Whitney, based on distribution, for statistical analysis. A Fisher's exact test was performed on binary data. A p-value <0.05 was considered statistically significant.

## Results

**Fig 1:**
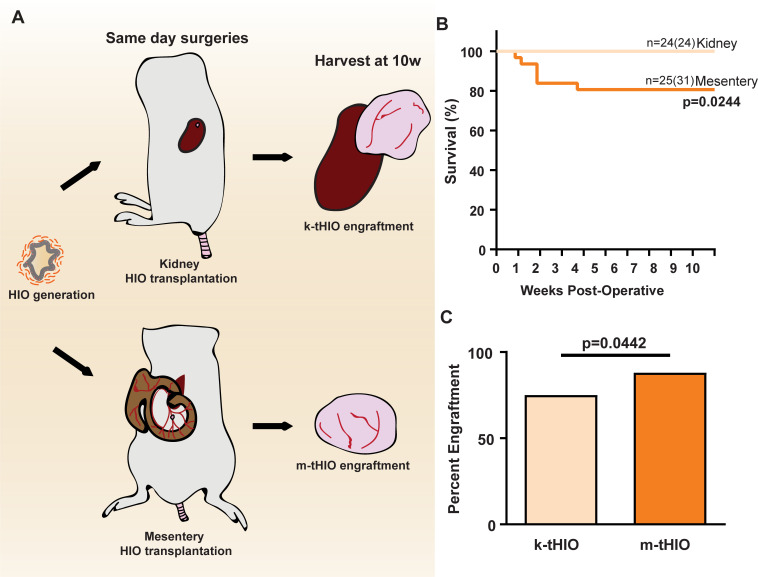
Transplantation into the RSS results in greater survival, but lower engraftment rates than the mesentery. (A) Experimental design schematic. (B) Kaplan-Meier curve associated with kidney and mesentery transplantation procedures (n = 24/24 and n = 25/31 respectively, p = 0.224). (C) Bar graph of engraftment rates at both transplantation sites (n = 17/24 and n = 22/25 respectively, p = 0.0442).

Three separate batches of HIOs generated from the H1 line were transplanted on d28 into either the RSS or mesentery of immunocompromised mice on the same day (Fig 1A). Ten weeks was allowed for engraftment and growth before harvesting and subsequent analysis. Mahe et al., have previously described both surgical procedures in detail [6]. Briefly, the RSS transplantation utilizes a silk thread to keep the kidney elevated. A pocket is created in the kidney capsule, the HIO inserted, and pushed along the renal surface deeper into the pocket to secure it within the RSS (S1A Fig). The mesentery transplantation utilizes octyl/butyl cyanoacrylate adhesive glue, which is applied to the mesentery, the HIO is then seeded atop the adhesive and allowed to cure (S1B Fig). Survival for RSS transplantation was significantly higher than in the mesentery (100% vs. 80.6%, n = 24/24 and n = 25/31 respectively, p = 0.0244; Fig 1C). However, engraftment was significantly lower in the RSS when compared to mesentery (75% vs. 88%, n = 17/24 and n = 22/25 respectively, p = 0.0442; Fig 1D).

**Fig 2:**
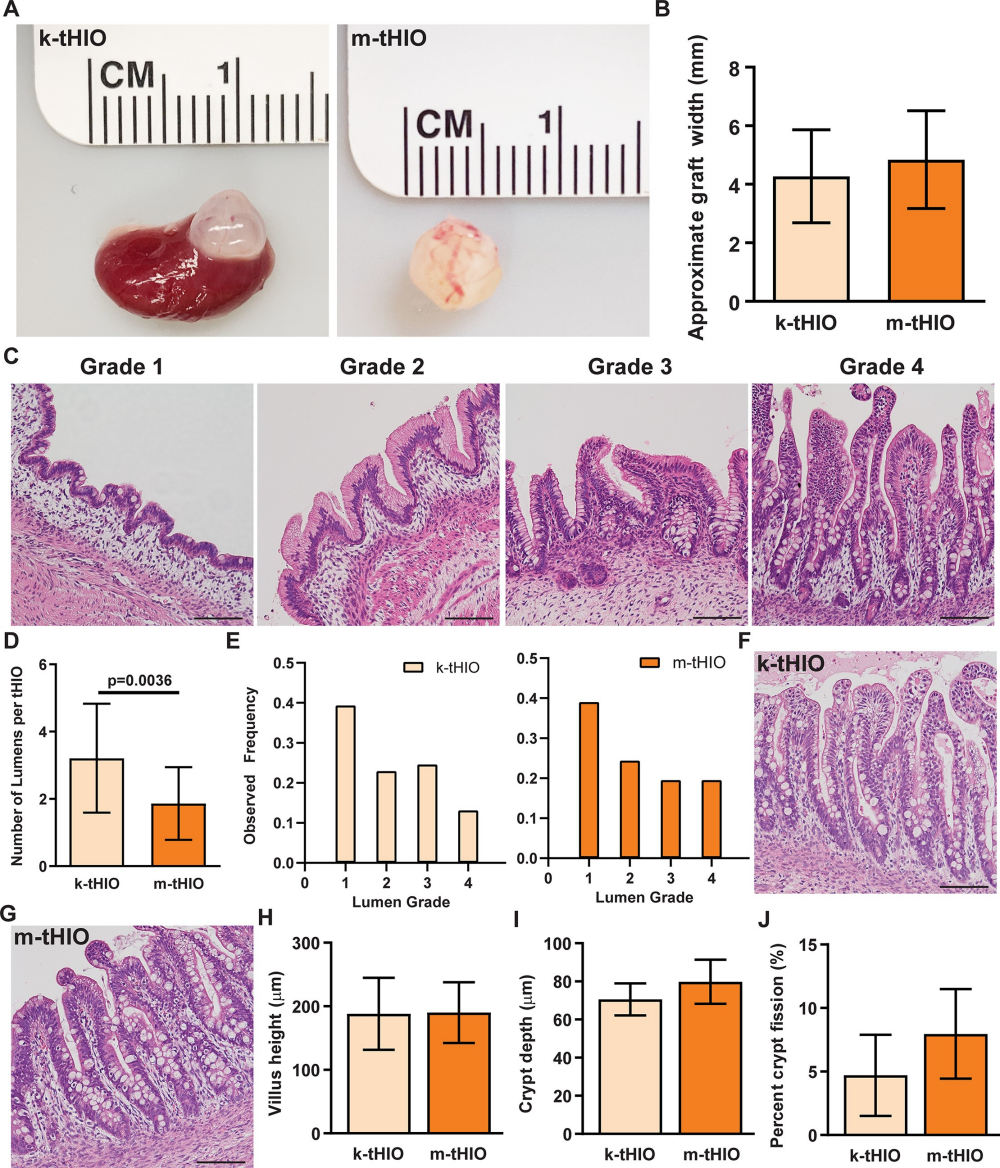
Grafts harvested from both sites had similar physical characteristics. (A) Gross images of representative k-tHIO (left) and m-tHIO (right). (B) Bar graph of the approximate graft width for k-tHIO and m-tHIO (n = 17 and n = 22, respectively; p = n.s.). (C) Representative images of hematoxylin and eosin stained sections of HIO Grades 1–4. Epithelial architecture is enhanced from Grades 1–4. Scale bars = 50 μm. (D) Bar graph of the number of lumens in a 2D cross-section of k-tHIO and m-tHIO (n = 17 and n = 22, respectively; p = 0.0036). (E) Histogram of lumen grades observed within k-tHIO (left) and m-tHIO (right). (n = 61 and n = 41, respectively). (F) Representative Grade 4 image of hematoxylin and eosin stained section of k-tHIO. Scale bar = 50 μm. (G) Representative Grade 4 image of hematoxylin and eosin stained section of m-tHIO. Scale bar = 50 μm. (H) Bar graph of average villus height in k-tHIO and m-tHIO (n = 6 and 9, respectively; p = n.s.). (G) Bar graph of average crypt depth in k-tHIO and m-tHIO (n = 6 and n = 9, respectively; p = n.s.). (H) Bar graph of crypt fission in k-tHIO and m-tHIO (n = 6 and n = 9, respectively; p = n.s.). H-J considered lumens of Grades 3 and 4.

Upon harvest at 10 weeks, kidney transplanted HIOs (k-tHIOs) and mesentery transplanted HIOs (m-tHIOs) appeared to be of similar size (Fig 2A). Quantifying graft size revealed no significant difference between k-tHIOs and m-tHIOs (Fig 2B). Observing hematoxylin and eosin stained sections revealed multiple lumens in some grafts and epithelial development to varying degrees in different tHIO lumens. To best describe the heterogeneity observed, four distinct grades were established: Grade 1, marked by a lack of crypt and villus structures; Grade 2, marked by the emergence of deep epithelial folding; Grade 3, marked by the presence of villus-like projections and crypts; and Grade 4, marked by elongated crypt-villus architecture (Fig 2C). Each tHIO lumen was quantified and assigned a grade. k-tHIOs presented with significantly more lumens than m-tHIOs (3.211 vs. 1.864, n = 17 and n = 22 respectively, p = 0.0036; Fig 2D). When examining the relative frequency of luminal grades present in k-tHIO and m-tHIO, no significant differences were observed (Fig 2E). Additionally, luminal grades were not found to correlat with graft size. Morphometric quantifications were performed on hematoxylin and eosin stained sections of Grades 3 and 4 to gain insight into potential differences in epithelial surface area (Fig 2F and 2G). Villus height and crypt depth were similar between groups (Fig 2H and 2I). Crypt fission, an indirect measure of stem cell expansion, was also similar between groups (Fig 2J) [9].

**Fig 3:**
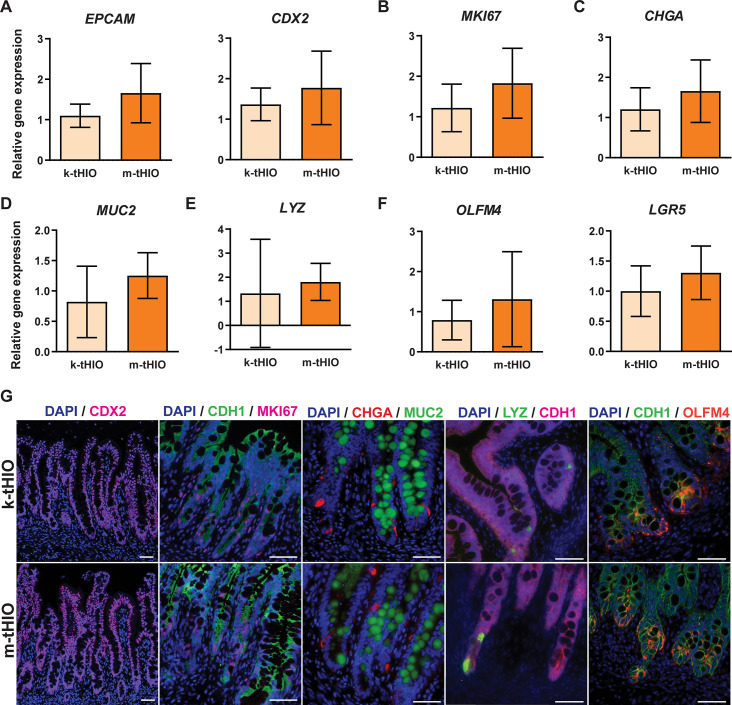
Grafts harvested from both sites developed secretory epithelial lineages. (A) Relative gene expression in k-tHIO and m-tHIO for epithelial development by EPCAM and CDX2. (B) Relative gene expression in k-tHIO and m-tHIO for proliferation by MKI67. (C) Relative gene expression in k-tHIO and m-tHIO for EECs by CHGA. (D) Relative gene expression in k-tHIO and m-tHIO for goblet cells by MUC2. (E) Relative gene expression in k-tHIO and m-tHIO for Paneth cells by LYZ. (F) Relative gene expression in k-tHIO and m-tHIO for the stem compartment by OLFM4 and LGR5. (A-G) For k-tHIO, n = 7 and for m-tHIO n = 5; p = n.s. for all. (G) Protein level expression, by immunofluorescence, in k-tHIO and m-tHIO associated to transcripts observed in A-F. Scale bars = 50 μm.

Epithelial development was interrogated next. General epithelial marker Epithelial Cell Adhesion Marker (EPCAM) and intestinal specific epithelial marker Caudal Type Homeobox 2 (CDX2) were present in all samples at both the transcript and protein levels (Fig 3A–3G) [10]. Proliferation, indicated by Marker of Proliferation KI67 (MKI67), within the grafts was also present in both groups to similar degrees (Fig 3B–3G). Epithelial proliferation, determined by Cadherin 1 (CDH1) and MKI67 double positivity in immunofluorescence staining, followed the typical pattern expected within gastrointestinal epithelium and was highly concentrated in the crypt regions (Fig 3G) [11].

Next, specific secretory cell types were evaluated using accepted markers [12]. Enteroendocrine cell (EEC) presence was confirmed in both groups by Chromogranin A (CHGA) expression at the transcript and protein levels (Fig 3C and 3G). Goblet cell presence was established in both groups by Mucin 2 (MUC2) expression at the transcript and protein levels (Fig 3D and 3G). Paneth cell presence was confirmed in both groups by Lysozyme (LYZ) expression at the transcript and protein levels (Fig 3E and 3G). The stem cell compartment was interrogated by both Olfactomedin 4 (OLFM4), an anti-apoptotic protein, and Leucine Rich Repeat Containing G Protein-Coupled Receptor 5 (LGR5), a stem cell marker [13, 14]. OLFM4 was observed in both groups at the transcript and protein levels, and LGR5 at the transcript level only, due to lack of reliable antibody availability (Fig 3F and 3G).

**Fig 4:**
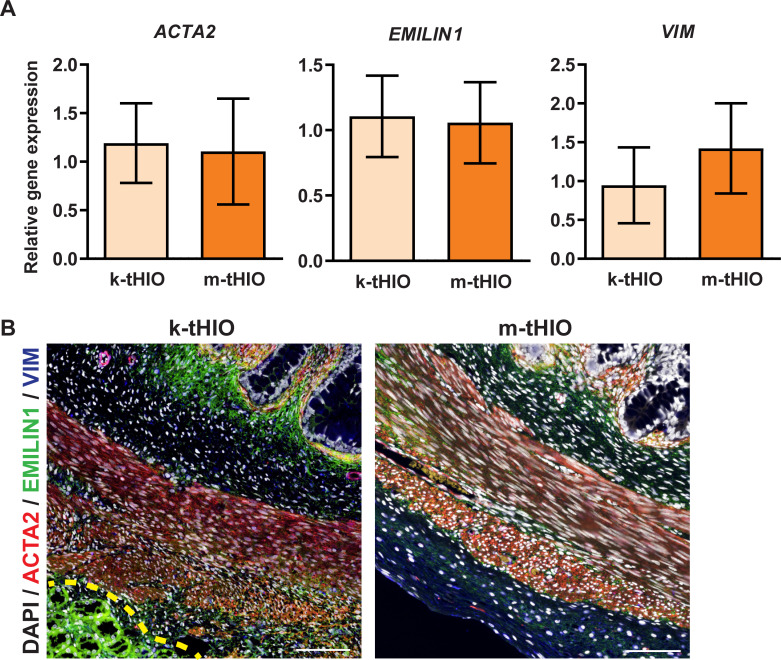
Grafts harvested from both sites developed mesenchyme. (A) Relative gene expression in k-tHIO and m-tHIO for mesenchymal components by ACTA2, EMILIN1, and VIM. For k-tHIO, n = 7 and for m-tHIO n = 5; p = n.s. for all. (B) Protein level expression, by immunofluorescence, in k-tHIO and m-tHIO associated to transcripts in A. Scale bars = 100 μm. Yellow dashed line indicates kidney margin.

Intestinal mesenchymal development was interrogated similarly. Actin Alpha 2, Smooth Muscle (ACTA2), a marker of muscle, was observed in both groups at the transcript and protein levels (Fig 4A and 4B). Elastin Microfibril Interfacer 1 (EMILIN1), an extracellular matrix glycoprotein we have found to be a pan-mesenchymal intestinal marker, along with Vimentin (VIM), a known fibroblast marker, were also observed in both groups at the transcript and protein levels (Fig 4A and 4B). Based on marker analysis, k-tHIO and m-tHIO appear to have developed comparable intestinal structures.

**Fig 5:**
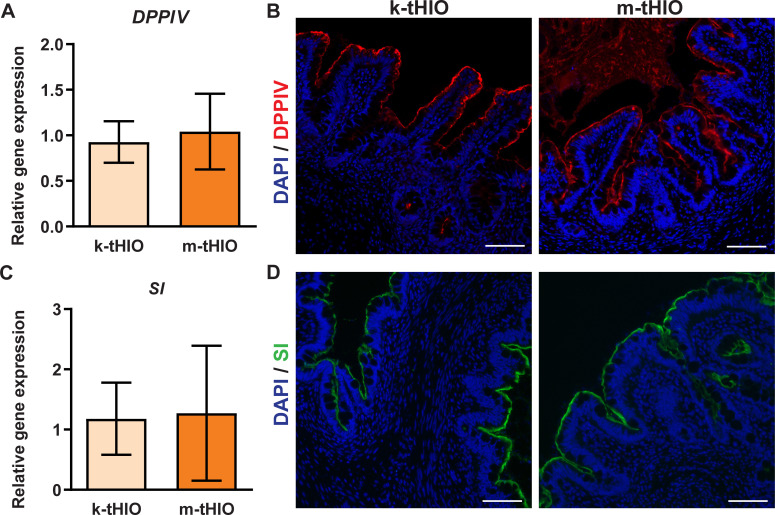
Grafts harvested from both sites expressed markers of carbohydrate digestion. (A) Relative gene expression of DPPIV in k-tHIO and m-tHIO. (B) Protein level expression, by immunofluorescence, in k-tHIO and m-tHIO associated to transcripts in A. Scale bars = 50 μm. (C) Relative gene expression of SI in k-tHIO and m-tHIO. (D) Protein level expression, by immunofluorescence, in k-tHIO and m-tHIO associated to transcripts in C. Scale bars = 50 μm. (A, C) For k-tHIO, n = 7 and for m-tHIO n = 5; p = n.s. for both.

To gain functional insight, enzymes involved in carbohydrate digestion were assessed. Dipeptidyl Peptidase 4 (DPPIV), active in glucose metabolism, was expressed at similar levels in both groups transcriptionally (Fig 5A) [15]. Protein level expression of DPPIV was robust in both groups (Fig 5B). Sucrase Isomaltase (SI), active in starch, sucrose and isomaltose metabolism, was expressed at similar levels in both groups transcriptionally (Fig 5C) [16]. Protein level expression of SI was also strong in both groups, suggesting that the capacity for carbohydrate digestion in k-tHIO and m-tHIO are similar with comparable enzyme expression.

## Discussion

HIO transplantation into both the RSS and mesentery is feasible and successful. Resultant tHIOs from both locations matured significantly when considering the *in vitro* starting material. The epithelial and mesenchymal architectures appeared similarly developed in k-tHIO and m-tHIO. The relative frequency in which luminal Grades 1–4 were observed was also similar between groups. Finally, as evidenced by transcript and protein level expression, secretory lineages were present, and muscle layers distinctly aligned throughout tHIOs in both groups.

Two major advantages of RSS transplantation are that proficiency in performing the technique is easily achieved and no mortality was observed. This model is ideal for developmental intestinal biology studies. Mice undergoing mesentery transplantation had a significantly increased mortality rate when compared to RSS. This cannot be attributed to anesthesia exposure, as the operative time was not extended for mesenteric versus RSS transplantation. We believe that other technical factors impacted the increased mortality rate during the first two weeks, as the procedure involves manipulating the bowel and delicate murine mesentery. Mortality beyond two weeks was found to result from intestinal obstruction due to the tHIO’s engraftment. Although more challenging and with a higher mortality, this model provides a physiological anatomic engraftment site important for functional in vivo studies.

An advantage of mesenteric transplantation is that the grafts consistently present with a dominant lumen, while, on average, k-tHIOs have significantly more lumens per tHIO. The presence of multiple lumens within k-tHIOs may be attributed to the introduction of mechanical disruption unique to the RSS transplantation process itself. Random septum formation seems unlikely, considering an individual d28 HIO with a single lumen is transplanted. During insertion within the renal capsule pocket, sliding the HIO along the kidney to secure it, and the added pressure of the enclosed space could all potentially damage or disrupt the HIO’s lumen [6]. As a result, tissue repair processes may produce multiple lumens.

The presence of one dominant lumen can be critical for downstream applications of tHIOs. For example, we previously incorporated strain exposure into the generation of tHIOs by implanting encapsulated springs within their lumen to induce maturation and enterogenesis [4]. This would not have been practical if the majority of tHIOs were multicystic. This will hold true for additional manipulations including performing intraluminal injections or placing pumps. Looking beyond basic science applications, when considering translational uses for HIO technologies, the presence of a well-developed intestinal structure with a single lumen will be crucial in recapitulating intestinal anatomy. Our previous work has demonstrated that tHIOs possess a functional epithelial barrier [3, 4], the ability to uptake peptides [3], and the ability to contract in a peristaltic-like movement when provided with an enteric nervous system [17]. Thus, tHIOs possess the capacity to eventually be used for therapeutic purposes. However, this requires generation of tissue with a single, well developed lumen. Transplantation into the mesentery may thus be more useful for potential therapeutic applications than transplantation into the RSS.

Additionally, tHIOs transplanted into the mesentery share a blood supply with the host gut. They can therefore be used to directly study drugs and nutrients in a clinically relevant fashion, as substances that m-tHIOs are exposed to will not undergo first-pass metabolism in the liver. tHIOs transplanted in the RSS cannot be used for this purpose, however, since they are exposed to the blood supply in the kidney.

Thus, tHIOs transplanted into the mesentery are likely more suitable for translational applications than tHIOs transplanted into the RSS.

## Conclusion

Ultimately, transplantation of HIOs into both the RSS and mesentery yield viable tissues that model the human gut. While k-tHIOs and m-tHIOs are roughly equivalent, one must strongly consider whether having a graft with a single lumen is important for their specific study and downstream uses at the cost of an increased mortality rate.

## Supporting information

S1 Fig Procedural images of RSS and mesentery HIO transplantations. (A) Photographs documenting an HIO transplantation into the RSS. A subcapsular pocket is created for HIO insertion. Once inserted, the HIO is pushed along the renal surface to secure it deeply within the capsule. Arrowheads indicate the subcapsular pocket. Black dashed lines outline the HIO. (B) Photographs documenting an HIO transplantation into the mesentery. Once a suitable location is identified for transplantation, a small drop of octyl/butyl cyanoacrylate adhesive glue is applied to the mesentery. Before the adhesive cures, an HIO is seeded upon it. Yellow dashed lines outline the adhesive pipette tip applicator. Black dashed lines outline the HIO.
(TIF)
