# Supplementary figures and images for: Evaluation of transplantation sites for human intestinal organoids
Source: PLoS One. 2020 Aug 27;15(8):e0237885. doi: 10.1371/journal.pone.0237885 (PMC7451647; doi:10.1371/journal.pone.0237885)

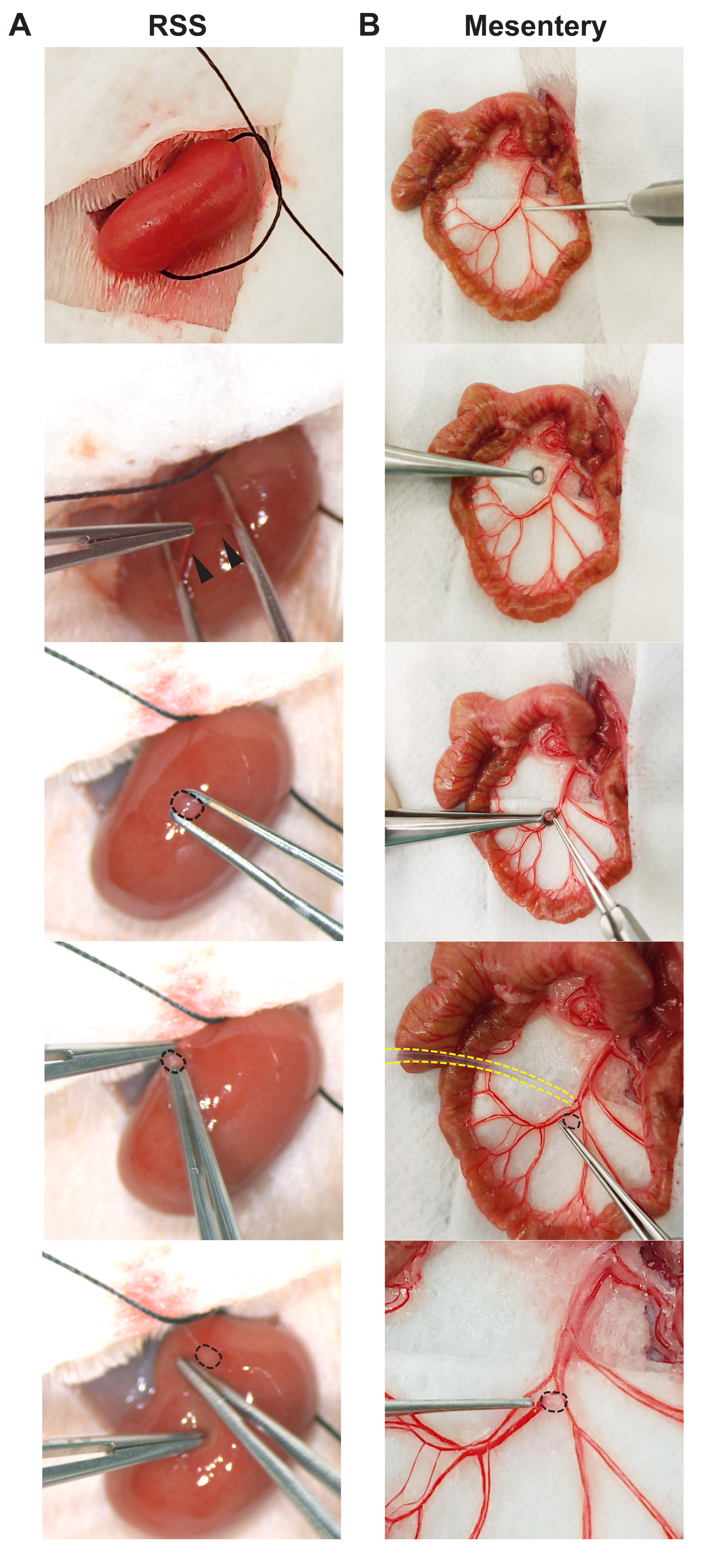

Supplement: S1 Fig — (A) Photographs documenting an HIO transplantation into the RSS. A subcapsular pocket is created for HIO insertion. Once inserted, the HIO is pushed along the renal surface to secure it deeply within the capsule. Arrowheads indicate the subcapsular pocket. Black dashed lines outline the HIO. (B) Photographs documenting an HIO transplantation into the mesentery. Once a suitable location is identified for transplantation, a small drop of octyl/butyl cyanoacrylate adhesive glue is applied to the mesentery. Before the adhesive cures, an HIO is seeded upon it. Yellow dashed lines outline the adhesive pipette tip applicator. Black dashed lines outline the HIO. (TIF) [file pone.0237885.s001.tif]
